# Coding-complete genome sequence of a GI-13 infectious bronchitis virus from commercial chicken in India

**DOI:** 10.1128/mra.01142-24

**Published:** 2025-01-27

**Authors:** Henry M. Kariithi, Jeremy D. Volkening, Claudio L. Afonso, Mohamed Helmy, Pushparaj P. Chaudhari, Eduardo L. Decanini

**Affiliations:** 1 Biotechnology Research Institute, Kenya Agricultural and Livestock Research Organization, Nairobi, Kenya; 2 BASE2BIO, Oshkosh, Wisconsin, USA; 3 Boehringer Ingelheim Animal Health IMETA, Dubai, UAE; 4 Boehringer Ingelheim India Pvt. Ltd, Mumbai, India; Katholieke Universiteit Leuven, Leuven, Belgium

**Keywords:** infectious bronchitis virus, lineage, GI-13, recombination event, polymorphism, indel

## Abstract

Infectious bronchitis virus (IBV) causes a highly contagious, acute upper respiratory disease in chickens characterized by nasal discharge, coughing, and rales. Here, the complete genome sequence of a recombinant GI-13 IBV strain ck/IN/A2332039-001/24 was sequenced from a choanal sample of a commercial broiler chicken in India using nontargeted next-generation sequencing.

## ANNOUNCEMENT

Direct-nontargeted metagenomic next-generation sequencing, which simultaneously identifies and genotypes complex pathogens (1), frequently identified infectious bronchitis virus (IBV; family *Coronaviridae* [2]) during a project on epidemiological mapping of pathogens and enhancing biosecurity in commercial chickens in India, Middle-East, Turkey, and Africa (IMETA) region. The globally important IBVs comprise of 36 lineages within eight genotypes (GI―GVIII) and unclassified inter-lineage variants (3). We report here a complete genome sequence of IBV from West Bengal, India.

Choanal swabs from 50 13-day-old asymptomatic broilers were pooled in 2-mL Kylt Swab buffer and spotted on AniCard (NGS Sampling Kit; SAN Group Biotech GmbH; Emstek, Germany). Total RNA was extracted using Kylt RNA/DNA Purification kit followed by proprietary in-house removal of host-specific DNAs and paired-end sequencing (2 × 150 bp; Illumina MiSeq 300-cycle Reagent Kit v2) at SAN Group Biotech. Using default parameters, raw reads (*n* = 458,051) were adapter-/quality-trimmed using Trim Galore v1.6.10 (-q 8), host-filtered using BBTools bbduk 39.01, assembled using MEGAHITv1.2.9 (4), and variants/alleles frequency analyses (> 10% and SB <30) using LoFreq v 2.1.5 and bcftools v 1.19. The *de novo*-assembled consensus sequences were annotated using Geneious Prime v2024.0.7 (5) and aligned with published IBV sequences using MAFFT v7.511 (6) for phylogenetics (7). Recombination events were analyzed using RDP4 v 4.101 (8).

The identified 10,667 IBV-specific read pairs (BLASTn-based) were *de novo*-assembled into a consensus genome sequence (one contiguous contig; median coverage depth of 116×) of 27,599 nucleotide (nt) in length (excluding poly-(A) tail) with a 38.2% GC content. Open reading frames of this strain (ck/IN/A2332039-001/24) have typical avian coronavirus organization (5′UTR-[Rep1ab-S-3a/3b-E-M-4a/4c-5a/5b-N-6b]−3′UTR), including conserved features of structural proteins (S, E, M, and N), cleavage sites and lengths of nsp2-16, the primary S1/S2 cleavage motif (amino acid residues ^536^**R**L**RR**↓**S** ^540^), and *N*-linked glycosylation sites (*n* = 27) of its 3,495-nt-long S gene (2, 9). Table 1 shows polymorphisms present in the coding regions of Rep1ab, S, 3a, and 6b genes.

**TABLE 1:** Polymorphism (eight nonsynonymous substitutions and one indel) present in the consensus genome sequence of the IBV GI-13 strain ck/IN/A2332039-001/24 identified in this study^*d*^

Gene^*a*^	Consensus sequence			Variant sequence		Coverage depth	Variant frequency	Coding effect^*c*^
	Position^*b*^	Codon	Amino acid residue	Codon	Amino acid residue			
Rep1ab (nsp1/2 [ZF-MF])	13,897	CTT	Leu	**T**TT	Phe	129	24%	SNP (nonsynonymous substitution)
Rep1ab (nsp11/12 [RdRp])	14,298	GCC	Ala	GC**A**	Cys	170	18.80%	SNP (nonsynonymous substitution)
Rep1ab (nsp13 [Hel])	15,153	GTA	Tyr	GT**G**	Val	91	13.20%	SNP (nonsynonymous substitution)
Rep1ab (nsp14 [ExoN])	17,619	TAT	Tyr	TA**C**	Val	81	37%	SNP (nonsynonymous substitution)
Spike (S)	262	TCT	Ser	**C**CT	Pro	170	15.90%	SNP (nonsynonymous substitution)
	356	CAA	Gln	C**C**A	Pro	92	19.60%	SNP (nonsynonymous substitution)
	863	GAC	Asp	G**G**C	Gly	76	18.40%	SNP (nonsynonymous substitution)
3a	83―85	GGT-A	Val	G**---**	-	104	17.30%	Indel (disruptive inframe deletion of 3 nt)
6b	181	GTG	Val	**A**TG	Met	332	21.10%	SNP (nonsynonymous substitution)

^*a*^ ExoN, exonuclease; Hel, helicase; nsp, nonstructural peptides; RdRp, RNA-dependent RNA polymerase; Rep1ab, replicase complex 1ab; ZF-MF, zinc-finger multifunctional protein.

^*b*^ Positions are reference to the start codon of the CDS of the genes.

^*c*^ SNP, single nucleotide polymorphism.

^*d*^ Alternate nucleotides in the codons of the minor variant compared to the consensus sequence are highlighted in bold and underlined text.

**Fig 1:**
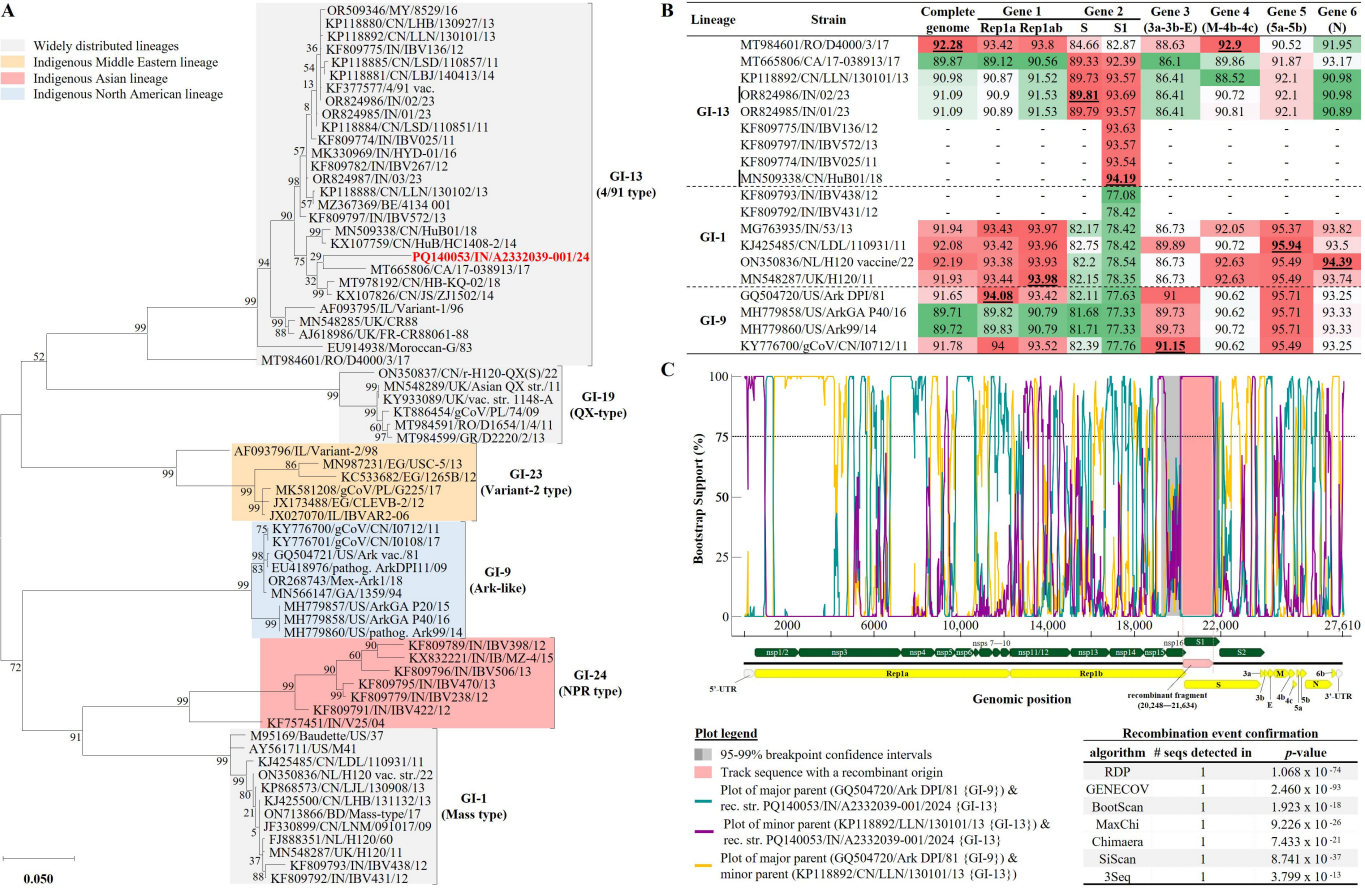
(**A**) Phylogenetic relationship of the IBV GI-13 strain ck/IN/A2332039-001/24 identified in this study (highlighted in bold red font) with a selection of other IBVs based on full-length S1 nt sequences. The tree was constructed using maximum likelihood method and GTR model (MEGA v 11.0.13; 1,000 replicates) involving 68 sequences and 1601 positions. (**B**) Similarities (% nt identities) of genome sequence and gene coding regions (CDS) of the six IBV genes of strain ck/IN/A2332039-001/24 identified in this study with GI-1, GI-9, and GI-13 viruses. The color gradient code (from red-white-green) indicate highest (dark red) to lowest (green) nt identities for each of the genes. bold underlined numbers and dash (“-”) indicate highest identities and unavailable sequences for each CDS, respectively. (**C**) Recombination event identified and confirmed by seven out of nine RDP4 algorithms (bottom right Table) (8) in the S1 sequence of strain ck/IN/A2332039-001/24 with a GI-9 strain GQ504720/Ark DPI/81 and GI-13 strain KP118892/LLN/130101/13 as the predicted major and minor parents, respectively. The recombination event has breakpoints beginning 74 nt upstream of the S gene (genomic position 20,248) and ending at nt position 21,634, producing a recombinant fragment of 1,387 bp in length, which covers 79.3% (1,283/1,617 nt) of the S1 gene. Sequence names include GenBank accession numbers, two-letter abbreviated country of origin, isolate name, and year of sample collection/identification.

The complete genome of ck/IN/A2332039-001/24 is most similar to a Romanian GI-13 (4/91-type) strain D4000/3/17 (92.3% nt identity) but phylogenetically distinct from other Indian 4/91 viruses (Fig. 1A and B). Recombinant nature of the Indian strain was suggested by highest similarities of its spike region to 4/91 viruses and genome backbone (nonspike region) to GI-1 (Mass-type) and GI-9 (Ark-type) viruses (Fig. 1B). This was confirmed (statistically supported by seven RDP4 algorithms) by detection of a 1,387-bp-long recombinant fragment in its S1 sequence (Fig. 1C). An Ark-type is the closest relative of the major parent (strain closest to the sequence surrounding the recombinant region; 92.6% nt identity), and 4/91-type the minor parent (strain closest to the recombinant fragment; 96.8% nt identity) of the Indian strain. Use of Mass- and 4/91-type and absence of Ark-type vaccines in India suggest strain ck/IN/A2332039-001/24 recombined and eventually diverged from its parental strains long before its detection. Although not supported by variant frequencies (Table 1), the possibility of a mixed IBV infection cannot be ruled out.

## Data Availability

Complete genome sequence of the IBV strain ck/IN/A2332039-001/24 reported in this paper has been deposited in GenBank (accession number PQ140053), and the raw data are available in the SRA (run accession number SRR30058295, BioSample number SAMN42953685, and BioProject number PRJNA1142602).

## References

[1] Afonso CL, Afonso AM. 2023. Next-generation sequencing for the detection of microbial agents in avian clinical samples. Vet Sci 10:690. doi:10.3390/vetsci10120690 38133241 PMC10747646

[2] Woo PCY, de Groot RJ, Haagmans B, Lau SKP, Neuman BW, Perlman S, Sola I, van der Hoek L, Wong ACP, Yeh S-H. 2023. ICTV virus taxonomy profile: Coronaviridae 2023. J Gen Virol 104:001843. doi:10.1099/jgv.0.001843 PMC12135074 37097842

[3] Valastro V, Holmes EC, Britton P, Fusaro A, Jackwood MW, Cattoli G, Monne I. 2016. S1 gene-based phylogeny of infectious bronchitis virus: an attempt to harmonize virus classification. Infect Genet Evol 39:349–364. doi:10.1016/j.meegid.2016.02.015 26883378 PMC7172980

[4] Li D, Liu C-M, Luo R, Sadakane K, Lam T-W. 2015. MEGAHIT: an ultra-fast single-node solution for large and complex metagenomics assembly via succinct de Bruijn graph. Bioinformatics 31:1674–1676. doi:10.1093/bioinformatics/btv033 25609793

[5] Kariithi HM, Volkening JD, Leyson CM, Afonso CL, Christy N, Decanini EL, Lemiere S, Suarez DL. 2022. Genome sequence variations of infectious bronchitis virus serotypes from commercial chickens in Mexico. Front Vet Sci 9:931272. doi:10.3389/fvets.2022.931272 35903135 PMC9315362

[6] Katoh K, Standley DM. 2013. MAFFT multiple sequence alignment software version 7: improvements in performance and usability. Mol Biol Evol 30:772–780. doi:10.1093/molbev/mst010 23329690 PMC3603318

[7] Tamura K, Stecher G, Kumar S. 2021. MEGA11: molecular evolutionary genetics analysis version 11. Mol Biol Evol 38:3022–3027. doi:10.1093/molbev/msab120 33892491 PMC8233496

[8] Martin DP, Murrell B, Golden M, Khoosal A, Muhire B. 2015. RDP4: detection and analysis of recombination patterns in virus genomes. Virus Evol 1:vev003. doi:10.1093/ve/vev003 27774277 PMC5014473

[9] Ayres GRR, Brandão PE. 2016. Avian coronavirus main replicase enzymes at a glance. Br J Virol 3:161–165. doi:10.17582/journal.bjv/2016.3.6.161.165

